# An improved method for the effect estimation of the intermediate event on the outcome based on the susceptible pre-identification

**DOI:** 10.1186/s12874-021-01378-8

**Published:** 2021-09-21

**Authors:** Haixia Hu, Ling Wang, Chen Li, Wei Ge, Jielai Xia

**Affiliations:** Department of Health Statistics, Faculty of Preventive Medicine, Air Force Medical University, No.169 Changle West Road, Xi’an, 710032 Shaanxi China

**Keywords:** Time-varying covariate, Mixture cure model, Landmark method, Extended Cox regression, Residual time distribution

## Abstract

**Background:**

In follow-up studies, the occurrence of the intermediate event may influence the risk of the outcome of interest. Existing methods estimate the effect of the intermediate event by including a time-varying covariate in the outcome model. However, the insusceptible fraction to the intermediate event in the study population has not been considered in the literature, leading to effect estimation bias due to the inaccurate dataset.

**Methods:**

In this paper, we propose a new effect estimation method, in which the susceptible subpopulation is identified firstly so that the estimation could be conducted in the right population. Then, the effect is estimated via the extended Cox regression and landmark methods in the identified susceptible subpopulation. For susceptibility identification, patients with observed intermediate event time are classified as susceptible. Based on the mixture cure model fitted the incidence and time of the intermediate event, the susceptibility of the patient with censored intermediate event time is predicted by the residual intermediate event time imputation. The effect estimation performance of the new method was investigated in various scenarios via Monte-Carlo simulations with the performance of existing methods serving as the comparison. The application of the proposed method to mycosis fungoides data has been reported as an example.

**Results:**

The simulation results show that the estimation bias of the proposed method is smaller than that of the existing methods, especially in the case of a large insusceptible fraction. The results hold for small sample sizes. Besides, the estimation bias of the new method decreases with the increase of the covariates, especially continuous covariates, in the mixture cure model. The heterogeneity of the effect of covariates on the outcome in the insusceptible and susceptible subpopulation, as well as the landmark time, does not affect the estimation performance of the new method.

**Conclusions:**

Based on the pre-identification of the susceptible, the proposed new method could improve the effect estimation accuracy of the intermediate event on the outcome when there is an insusceptible fraction to the intermediate event in the study population.

**Supplementary Information:**

The online version contains supplementary material available at 10.1186/s12874-021-01378-8.

## Background

In the context of follow-up studies, some patients may experience the intermediate event before the occurrence of the outcome of interest. The instances of intermediate events include the occurrence of the objective disease response or adverse events, the change in a biomarker, or the initiation of a subsequent or secondary treatment [1]. Like baseline variables, the intermediate event could change the risk of the outcome but in the form of a time-varying covariate. More and more researchers are interested in the effect of the time-varying intermediate event [2, 3]. For simplicity and differentiation, we express the time-varying intermediate event as “event” and the event of interest as “outcome” hereinafter. Rather than being determined at entry as in randomized controlled trials, the group of each patient is based on the whole follow-up in studies of time-varying intermediate events. The time from entry to the intermediate event varies from patient to patient. Some patients may die or drop out of the trial before the occurrence of the intermediate event and they are classified into the event-free group as a consequence. For patients who have experienced the intermediate event, there is a period of time during which the outcome, such as death, did not happen. This period of time is classified into the event group in traditional survival analysis, which is in favor of the event. Furthermore, patients who have or have not experienced the intermediate event may be heterogeneous. The outcome is more likely to happen or it happens earlier to patients in the event-free group. Therefore, bias in the effect estimation of the time-varying intermediate event is incurred using the traditional survival analysis, which is called guarantee time bias or immortal time bias [4, 5].

Suissa [6] quantified the magnitude of the guarantee time bias under different survival distributions and various study designs. To deal with the guarantee time bias, Mantel and Byar method [7–9], also called extended Cox regression, analyzes the time-varying intermediate event data by grouping with the person-time instead of patients. Patients who have experienced the time-varying intermediate event are classified into the event-free group before the occurrence of the intermediate event and classified into the event group after the occurrence of the intermediate event. The extended Cox regression has been proved to provide unbiased estimates [4] and is recommended as a method to eliminate the guarantee time bias [6]. Cho et al. [5] recommended the extended Cox regression for analyzing the cumulative and long-term drug exposure. The limitation of the extended Cox regression is its incapability to visualize the survival curve for each group so the effect of the time-varying intermediate event is not intuitively clear. Anderson et al. [10] proposed the landmark method to eliminate guarantee time bias. They suggested analyzing the data of patients who have survived to the chosen landmark time and classifying the patients into either the event group or the event-free group based on their intermediate event status at the landmark time without considering the possible shift after that. The landmark method performs well when the effect is small [1] though in a less powerful manner because of the conditional nature. The landmark method has been widely applied to the dynamic prediction for time-to-event data or other data types [11–13]. Recently, a pooled summary analysis of several landmarks, i.e., the landmark supermodel, has been advocated to smooth the effect of the time-varying intermediate event [14, 15]. The naïve method [16] and exclusion method [6] are also alternative methods to handle guarantee time bias. But both of them are not recommended based on the results of simulation studies [4].

Despite extensive works focusing on eliminating the bias when estimating the effect of the time-varying intermediate event, an insusceptible fraction [17–22] to the intermediate event in population has not been considered in existing literature [1, 4, 6]. For instance, in studies that estimate the effect of the acute graft-versus-host disease (aGVHD) on the relapse or death of patients following hematopoietic cell transplantation, some of the patients would never experience the aGVHD, i.e., they are not susceptible or immune to the aGVHD [23]. For existing methods, i.e., the extended Cox regression and landmark methods, the patients insusceptible to the intermediate event would be classified into the event-free group since the intermediate event could not be observed. However, the hazards of the outcome are different in patients who are susceptible but have not experienced the intermediate event and patients who are insusceptible to the intermediate event. The mix of insusceptible patients would change the hazard of the outcome in the event-free group, leading to the bias in the effect estimation of the time-varying intermediate event further.

Regarding the insusceptible/cure fraction in survival analysis, most previous researches concentrate on the cure fraction to the outcome (i.e., dependent variables) [24–26] instead of the insusceptible fraction to the intermediate event (i.e., independent variables). The logistic regression model (LRM) has been widely used to identify the cure fraction to the outcome [20, 24, 27]. Lee’s study [23] has taken into account the insusceptible fraction to the intermediate event. They derived the risk prediction for the time-varying intermediate event (aGVHD in their study) via a novel multi-state model which was built on Conlon’s [28] multi-state cure model. Both Lee’s and Conlon’s models estimated the risk function of the outcome (death in both studies), the time-varying intermediate event (aGVHD in Lee’s study and recurrence in Conlon’s study), and the transition of the intermediate event to the outcome. However, the effect of the time-varying intermediate event on the outcome has not been taken into account in their models.

In this paper, we aim to estimate the effect of the time-varying intermediate event on the outcome when there is an insusceptible fraction to the intermediate event. We propose a new effect estimation method in which the susceptible subpopulation pre-identification is newly considered. Patients who have experienced the intermediate event are susceptible to the event without a doubt. While the susceptibility of the patient with censored intermediate event time could be predicted based on the following two considerations. 1) There are dissimilarities between the susceptible and insusceptible subpopulations, such as the distribution of covariates that influence the susceptibility to the intermediate event. Note that the occurrence of most intermediate events is dependent on the characteristics of the patient but not the external environment. Some endogenous covariates, such as the severity of the illness and the biomarker level, make the patient more prone to the occurrence of the intermediate event. 2) The time to the intermediate event can partly reflect the susceptibility of the patient. For example, patients who have experienced neither the outcome nor the intermediate event for a long time are more likely to be insusceptible to the intermediate event. Since the information of the incidence and time of the time-varying intermediate event is combined by the mixture cure models [29], we propose to predict the susceptibility of patients via the residual time distribution [30] of the intermediate event based on the mixture cure model. The patient with censored intermediate event time is more likely to be insusceptible to the intermediate event when his/her residual time of the intermediate event is incalculable using the event time distribution of the susceptible subpopulation. Then, the extended Cox regression and landmark methods are employed to estimate the effect of the time-varying intermediate event in the identified susceptible population. The proposed new method hopes to reduce the estimation bias of existing methods by mitigating the interference from the insusceptible subpopulation and conducting the effect estimation in the right population.

## Methods

To estimate the effect of the time-varying intermediate event when there is an insusceptible fraction to it in the study population, we propose an improved method in which the susceptible subpopulation pre-identification is newly considered. There are three steps in the new method as summarized in Table 1: 1) fit the incidence and time of the intermediate event with the mixture cure model; 2) pre-identification of the susceptible subpopulation; 3) effect estimation based on the identified susceptible subpopulation.

**Table 1:** Three steps of the proposed effect estimation method based on the susceptible pre-identification

**Step 1:** Fit the incidence and time of the intermediate event with the mixture cure model. Based on the intermediate event data, maximize the following likelihood function to obtain the estimates of **β**_**e**_ and **γ**, $L\left({\boldsymbol{\upbeta}}_{\mathbf{e}},\boldsymbol{\upgamma} \right)=\prod \limits_{i=1}^N\left\{{\left[\pi \left({\mathbf{x}}_i\right)f\left({t}_{ei}|s=1,{\mathbf{x}}_i\right)\right]}^{\delta_{ei}}\times {\left[1-\pi \left({\mathbf{x}}_i\right)+\pi \left({\mathbf{x}}_i\right)S\left({t}_{ei}|s=1,{\mathbf{x}}_i\right)\right]}^{1-{\delta}_{ei}}\right\}$ where *π*(**x**) = [1 + exp(−(*γ*_0_ + **γ**^*T*^**x**))]^−1^, $S\left({t}_e|s=1,\mathbf{x}\right)=\exp \left(-{\lambda}_e{t_e}^{v_e}\exp \left({{\boldsymbol{\upbeta}}_{\mathbf{e}}}^T\mathbf{x}\right)\right)$ and *f*(*t*_*e*_| *s* = 1, **x**) = *d*[1 − *S*(*t*_*e*_| *s* = 1, **x**)]/*dt*_*e*_.	
**Step 2:** Pre-identification of the susceptible subpopulation. (1) For patients that have experienced the intermediate event the susceptibility is *s* = 1, i.e., being susceptible to the intermediate event. (2) For patients with censored intermediate event time the susceptibility is *s* = 1 when ${u}_i>\frac{1-\pi \left({\mathbf{x}}_i\right)}{1-\pi \left({\mathbf{x}}_i\right)+\pi \left({\mathbf{x}}_i\right)S\left({C}_{ei}|s=1,{\mathbf{x}}_i\right)}$and *s* = 0, i.e., being insusceptible, when ${u}_i\le \frac{1-\pi \left({\mathbf{x}}_i\right)}{1-\pi \left({\mathbf{x}}_i\right)+\pi \left({\mathbf{x}}_i\right)S\left({C}_{ei}|s=1,{\mathbf{x}}_i\right)}$ where *u*_*i*_ is a random number from the uniform distribution *U*(0, 1).	
**Step 3:** Effect estimation based on the identified susceptible subpopulation. Based on the identified susceptible subpopulation, estimate the effect of the time-varying intermediate event which is quantified by *β*_*z*_. For the extended Cox regression method, *h*(*t*_*o*_| **x**, *z*(*t*_*o*_)) = *h*_0_(*t*_*o*_) exp(**β**_**o**_^*T*^**x** + *β*_*z*_*z*(*t*_*o*_)). For the landmark method, $h\left({t}_o|\mathbf{x},{z}_{t_{LM}}\right)={h}_0\left({t}_o\right)\exp \left({{\boldsymbol{\upbeta}}_{\mathbf{o}}}^T\mathbf{x}+{\beta}_z{z}_{t_{LM}}\right),$ for patients with *t*_*o*_ > *t*_*LM*_.	

### Step 1: fit the incidence and time of the intermediate event with the mixture cure model

Suppose a cohort composed of the susceptible and the insusceptible patients, the susceptible may experience the intermediate event sometime in follow-up while the insusceptible may never not. Assume there are *N* patients in the cohort. Let *r*(0 < *r* < 1) denote the proportion of the susceptible in the study population, *s* be an indicator denoting whether a patient is susceptible (*s* = 1) or insusceptible (*s* = 0), and *T*_*e*_ be the time to the time-varying intermediate event for the susceptible. Then the cumulative incidence function of the time-varying intermediate event at time *t*_*e*_ modeled by the mixture cure model is expressed as [31].

1 $$ F\left({t}_e|\mathbf{x},\mathbf{z}\right)=1-S\left({t}_e|\mathbf{x},\mathbf{z}\right)=1-\left[1-\pi \left(\mathbf{x}\right)+\pi \left(\mathbf{x}\right)S\left({t}_e|s=1,\mathbf{z}\right)\right] $$

where *π*(**x**) = *P*(*s* = 1| **x**) is the probability of being susceptible to the intermediate event for the patient with covariate vector of **x** = (*x*_1_, …, *x*_*g*_)^*T*^, *S*(*t*_*e*_| *s* = 1, **z**) is the probability that a susceptible patient with covariate vector of **z** = (*z*_1_, …, *z*_*j*_)^*T*^ has not experienced the intermediate event up to time *t*_*e*_. The vectors of **x** and **z** could be the same or different and we set them the same for ease of notation in the following parts. The LRM and Weibull distribution are used to model the susceptible probability and the time to the intermediate event for susceptible patients, respectively, as done in the literature [20, 22, 31, 32]. Specifically, the susceptible probability for the patient with covariate vector **x** is expressed as

2 $$ \pi \left(\mathbf{x}\right)=\frac{1}{1+\exp \left[-\left({\gamma}_0+{\boldsymbol{\upgamma}}^T\mathbf{x}\right)\right]} $$

and the probability that a susceptible patient with covariate vector **x** has not experienced the intermediate event up to time *t*_*e*_ is formulated as

3 $$ S\left({t}_e|s=1,\mathbf{x}\right)=\exp \left(-{\lambda}_e{t_e}^{v_e}\exp \left({{\boldsymbol{\upbeta}}_{\mathbf{e}}}^T\mathbf{x}\right)\right) $$

where **γ** and **β**_**e**_ are the coefficient vectors of the covariate vector **x**, *λ*_*e*_ and *ν*_*e*_ are the scale and shape parameters of the Weibull distribution, respectively. Suppose that the intermediate event time, the outcome time, and the administrative censoring time (i.e., the longest follow-up time) for the *i-*th (*i* = 1, …, *N*) patient are denoted by *T*_*ei*_, *T*_*oi*_, and *τ*, respectively, the observed intermediate event time *t*_*ei*_ = min(*T*_*ei*_, *T*_*oi*_, *τ*). For the sake of distinction, the subscript character “e” is used hereinafter for the time-varying intermediate event while the subscript character “o” for the outcome. For the insusceptible patient (*s* = 0), *T*_*ei*_ is supposed to be infinite and larger than *τ*. For the susceptible patient (*s* = 1), the occurrence of the intermediate event could be censored by both the outcome and the end of the follow-up. In other words, the intermediate event time might not be observed (*δ*_*ei*_ = 0) due to insusceptibility or censoring. Accordingly, the censored intermediate event time *t*_*ei*_ = *C*_*ei*_ = min(*T*_*oi*_, *τ*). Otherwise, the observed intermediate event time *t*_*ei*_ = *T*_*ei*_ and the censoring indicator *δ*_*ei*_ = 1. Based on the mixture cure model described in eq. (1), the likelihood of the observed data can be written as

4 $$ L\left({\boldsymbol{\upbeta}}_{\mathbf{e}},\boldsymbol{\upgamma} \right)=\prod \limits_{i=1}^N\left\{{\left[\pi \left({\mathbf{x}}_i\right)f\left({t}_{ei}|{s}_i=1,{\mathbf{x}}_i\right)\right]}^{\delta_{ei}}\times {\left[1-\pi \left({\mathbf{x}}_i\right)+\pi \left({\mathbf{x}}_i\right)S\left({t}_{ei}|{s}_i=1,{\mathbf{x}}_i\right)\right]}^{1-{\delta}_{ei}}\right\} $$

where *f*(*t*_*e*_| *s* = 1, **x**) = *d*[1 − *S*(*t*_*e*_| *s* = 1, **x**)]/*dt*_*e*_ is the probability density function (PDF) of the intermediate event for susceptible patients. The estimates of **γ** and **β**_**e**_ can be obtained by maximizing the likelihood in eq. (4) via the expectation-maximization (EM) algorithm. More details on the estimation and computation process can be found in Peng and Dear [21] and Sy and Taylor [20], which are not repeated here to avoid tedious descriptions.

The estimation process has been compiled to a SAS macro, named %PSPMCM, by Corbière and Joly [31]. In SAS macro %PSPMCM, the logit link in eq. (2) could be replaced by the probit link and the log-log link, and the Weibull distribution in eq. (3) could be replaced by the exponential, lognormal, log-logistic distributions, or the Cox model. The alternative link functions and distributions can be adopted when the LRM and the Weibull distribution do not fit the data well.

### Step 2: pre-identification of the susceptible subpopulation

Patients who have experienced the intermediate event are classified as susceptible. For patients with censored intermediate event time, the susceptibility is predicted based on the residual time distribution [30] of the intermediate event. Let *a*_*ei*_ be the residual time for the intermediate event after the censored intermediate event time *C*_*ei*_, where *C*_*ei*_ = min(*T*_*oi*_, *τ*) as aforementioned in Step 1. According to the mixture cure model, the conditional distribution of the intermediate event time for the *i*-th patient with censored intermediate event time is given by [30].

5 $$ P\left({T}_{ei}>{C}_{ei}+{a}_{ei}|{T}_{ei}>{C}_{ei}\right)=\frac{1-\pi \left({\mathbf{x}}_i\right)+\pi \left({\mathbf{x}}_i\right)S\left({C}_{ei}+{a}_{ei}|s=1,{\mathbf{x}}_i\right)}{1-\pi \left({\mathbf{x}}_i\right)+\pi \left({\mathbf{x}}_i\right)S\left({C}_{ei}|s=1,{\mathbf{x}}_i\right)} $$

where *P*(*T*_*ei*_ > *C*_*ei*_ + *a*_*ei*_| *T*_*ei*_ > *C*_*ei*_) ∈ (0, 1). We generate a random number *u*_*i*_ from the uniform distribution *U*(0, 1) for each patient with censored intermediate event time and set *P*(*T*_*ei*_ > *C*_*ei*_ + *a*_*ei*_| *T*_*ei*_ > *C*_*ei*_) = *u*_*i*_. Then, we have

6 $$ {a}_{ei}={S}^{-1}\left(\frac{u_i\left[1-\pi \left({\mathbf{x}}_i\right)+\pi \left({\mathbf{x}}_i\right)S\left({C}_{ei}|s=1,{\mathbf{x}}_i\right)\right]-\left[1-\pi \left({\mathbf{x}}_i\right)\right]}{\pi \left({\mathbf{x}}_i\right)}\right)-{C}_{ei}. $$

In eq. (6), *u*_*i*_[1 − *π*(**x**_*i*_) + *π*(**x**_*i*_)*S*(*C*_*ei*_| *s* = 1, **x**_*i*_)] − [1 − *π*(**x**_*i*_)] is supposed to be positive since *S*(*T*_*ei*_| *s* = 1, **x**) ∈ (0, 1). That is, $\frac{1-\pi \left({\mathbf{x}}_i\right)}{1-\pi \left({\mathbf{x}}_i\right)+\pi \left({\mathbf{x}}_i\right)S\left({C}_{ei}|s=1,{\mathbf{x}}_i\right)}<{u}_i$. With *u*_*i*_ being a random number from the uniform distribution *U*(0, 1), either of the following two conditions may occur to the *i*-th patient: 1) $\frac{1-\pi \left({\mathbf{x}}_i\right)}{1-\pi \left({\mathbf{x}}_i\right)+\pi \left({\mathbf{x}}_i\right)S\left({C}_{ei}|s=1,{\mathbf{x}}_i\right)}<{u}_i$ and *a*_*ei*_ could be calculated with eq. (6). That is, the patient may experience the intermediate event at *a*_*ei*_ after the censoring time *C*_*ei*_. Therefore, we identify the patient as susceptible. 2) $\frac{1-\pi \left({\mathbf{x}}_i\right)}{1-\pi \left({\mathbf{x}}_i\right)+\pi \left({\mathbf{x}}_i\right)S\left({C}_{ei}|s=1,{\mathbf{x}}_i\right)}\ge {u}_i$. In this case, the value of *u*_*i*_[1 − *π*(**x**_*i*_) + *π*(**x**_*i*_)*S*(*C*_*ei*_| *s* = 1, **x**_*i*_)] − [1 − *π*(**x**_*i*_)] is negative and the residual time for the intermediate event (*a*_*ei*_) is incalculable. In other words, *S*(*T*_*ei*_| *s* = 1, **x**) is not applicable to calculate the residual intermediate event time because the patient does not belong to the category of the susceptible, i.e., *s* ≠ 1. The patient is considered to be insusceptible to the intermediate event then. Viewed from another perspective, with *u*_*i*_ following the uniform distribution *U*(0, 1), there is $P\left(\frac{1-\pi \left({\mathbf{x}}_i\right)}{1-\pi \left({\mathbf{x}}_i\right)+\pi \left({\mathbf{x}}_i\right)S\left({C}_{ei}|s=1,{\mathbf{x}}_i\right)}\ge {u}_i\right)=\frac{1-\pi \left({\mathbf{x}}_i\right)}{1-\pi \left({\mathbf{x}}_i\right)+\pi \left({\mathbf{x}}_i\right)S\left({C}_{ei}|s=1,{\mathbf{x}}_i\right)}$. That is, the probability that a patient is classified as insusceptible is equal to the probability that he/she belongs to the insusceptible part 1 − *π*(**x**_*i*_), which is reasonable. To sum up, patients who have not experienced the intermediate event but with $\frac{1-\pi \left({\mathbf{x}}_i\right)}{1-\pi \left({\mathbf{x}}_i\right)+\pi \left({\mathbf{x}}_i\right)S\left({C}_{ei}|s=1,{\mathbf{x}}_i\right)}$ equal or greater than the random number *u*_*i*_ following *U*(0, 1) are classified as insusceptible. The other patients with censored intermediate event time are classified as susceptible conversely.

As above, patients with censored intermediate event time are classified into either the susceptible or the insusceptible according to whether the residual intermediate event time could be imputed. We call the proposed classification method the residual intermediate-event time imputation (RITI) method. The proposed RITI method incorporates the information of both the susceptible probability, i.e., *π*(**x**), and the intermediate event time distribution of the susceptible, i.e., *S*(*C*_*e*_| *s* = 1, **x**).

For comparison and completeness, we adopt the LRM to classify the patients with censored intermediate event time, since the LRM is a widely used model for the classification issue [33–35]. To be specific, the logistic regression part of the mixture cure model, i.e., *π*(**x**), is used to calculate the susceptible probability of the patient with censored intermediate event time. Then a random number following the Bernoulli distribution with the probability of *π*(**x**) is generated. The patient is classified as susceptible if the random number is one, and insusceptible on the contrary. Compared to the proposed RITI method, it is straightforward that the LRM only takes advantage of the incidence part of the mixture cure model, losing the information of the conditional survival function, i.e., *S*(*C*_*e*_| *s* = 1, **x**), when identifying the susceptible.

### Step 3: effect estimation based on the identified susceptible subpopulation

Based on the identified susceptible patients, we employ the extended Cox regression and landmark methods to estimate the effect of the intermediate event on the outcome. For the extended Cox regression, the hazard function of the outcome is expressed as

7 $$ h\left({t}_o|\mathbf{x},z\left({t}_o\right)\right)={h}_0\left({t}_o\right)\exp \left({{\boldsymbol{\upbeta}}_{\mathbf{o}}}^T\mathbf{x}+{\beta}_zz\left({t}_o\right)\right) $$

where *t*_*o*_ is the outcome time, *z*(*t*_*o*_), a time-varying variable, is the indicator for the occurrence of the intermediate event at time *t*_*o*_ with *z*(*t*_*o*_) = 1 for patients who have experienced the intermediate event and *z*(*t*_*o*_) = 0 otherwise, and **β**_**o**_ is the covariate coefficient vector. The baseline hazard function of Weibull distribution is used in this paper, i.e., ${h}_0\left({t}_o\right)={\lambda}_o{\nu}_o{t_o}^{\nu_o-1}$. For the landmark method with the landmark time *t*_*LM*_, the hazard function of the outcome is expressed as

8 $$ h\left({t}_o|\mathbf{x},{z}_{t_{LM}}\right)={h}_0\left({t}_o\right)\exp \left({{\boldsymbol{\upbeta}}_{\mathbf{o}}}^T\mathbf{x}+{\beta}_z{z}_{t_{LM}}\right),{t}_o>{t}_{LM} $$

where the intermediate event indicator ${z}_{t_{LM}}$ is not a time-varying variable as in eq. (7), it is a fixed value determined by the intermediate event status at the landmark time. Patients who have experienced the outcome before the landmark time are not included in the analysis. The effect of the intermediate event is quantified by the coefficient *β*_*z*_ in eqs. (7) and (8).

The details of the extended Cox regression and landmark methods are not elaborated here. Readers interested are referred to Mantel [7], Martinussen [36], and Therneau [8] for extended Cox regression method and are referred to Van Houwelingen [37], Anderson [10], and Dafni [38] for the landmark method.

## Results

We conducted Monte-Carlo simulations to assess the estimation performance of the proposed method in different scenarios, as described in later subsection “Simulation results”. As summarized in Table 2, we also investigated the estimation performances of other methods for comparison purposes. The methods included in simulations are as follows.

1. Existing methods that estimate the effect of the time-varying intermediate event based on the entire population (coded as exCox1 for the extended Cox regression and LM1 for the landmark method).
2. Control methods with the susceptible subpopulation identified by existing LRM (coded as exCox2 and LM2 when the effect was estimated via the extended Cox regression and the landmark method, respectively).
3. The proposed new methods with the susceptible subpopulation identified by the RITI method (coded as exCox3 and LM3 when the effect was estimated via the extended Cox regression and the landmark method, respectively).
4. Performance benchmark: existing methods that estimate the effect based on the real susceptible subpopulation (coded as exCox4 and LM4 when the effect was estimated via the extended Cox regression and the landmark method, respectively). It is necessary to take the methods exCox4 and LM4 into account to highlight the effect of the susceptible subpopulation pre-identification process. However, it is worth noting that it is impossible to obtain the results of these two methods in practice because the susceptibilities of patients with censored intermediate event time are unknown.

**Table 2:** Details of the methods investigated in the simulation study

Method Code	The susceptible pre-identification		Effect estimation method		Analysis set		
	LRM	RITI	exCox	LM	Entire population	Identified susceptible subpopulation	Real susceptible subpopulation
exCox1			✓		✓		
exCox2	✓		✓			✓	
exCox3		✓	✓			✓	
exCox4			✓				✓
LM1				✓	✓		
LM2	✓			✓		✓	
LM3		✓		✓		✓	
LM4				✓			✓

The proposed new methods were coded as exCox3 and LM3. Existing methods were coded as exCox1-exCox2 and LM1-LM2. The results of methods exCox4 and LM4 were served as the performance benchmark. Abbreviations: *exCox* extended Cox regression; *LM* landmark method; *LRM* logistic regression model; *RITI* residual intermediate-event time imputation

### Simulation setting

The data for the *i*-th patient in the simulation study included {*s*_*i*_, *t*_*ei*_, *δ*_*ei*_, *t*_*oi*_, *δ*_*oi*_, **x**_*i*_}, where *s*_*i*_ was the susceptible indicator with *s*_*i*_ = 1 for the susceptible and *s*_*i*_ = 0 for the insusceptible, **x**_*i*_ was the covariate vector and *t*_*ei*_, *δ*_*ei*_, *t*_*oi*_, *δ*_*oi*_ were the observed time and the censoring indicator for the intermediate event and the outcome, respectively, with *δ*_⋅*i*_ = 1 for uncensored data and *δ*_⋅*i*_ = 0 for censored data. Note that the intermediate event time could be censored by the occurrence of the outcome but not vice versa. Both the intermediate event time and the outcome time could be censored by the study termination. For illustration, we assumed no dropout in this paper.

#### Covariate vector

For the covariate vector **x** that influences the hazard of the intermediate event and the outcome, three scenarios were considered as follows.

Scenario (i): Four covariates *X*_1_-*X*_4_ following independent Bernoulli distributions with the probability of 0.1, 0.2, 0.3, and 0.5, respectively.

Scenario (ii): Four covariates *X*_1_-*X*_4_, where *X*_1_-*X*_2_ following independent Bernoulli distributions with the probability of 0.3 and 0.5, and *X*_3_-*X*_4_ following independent uniform distributions in (0,5) and (0,10), respectively.

Scenario (iii): Six covariates *X*_1_-*X*_6_, where *X*_1_-*X*_4_ being the same as the scenario (i) and *X*_5_-*X*_6_ following independent uniform distributions in (0,5) and (0,10), respectively. That is, scenario (iii) was the combination of scenarios (i) and (ii).

With the three scenarios, both the number and type of covariates have been taken into account.

#### Susceptibility

We generated random numbers with the LRM to simulate the population with a specified susceptible proportion. Assume all covariates affect the susceptibility, the probability of being susceptible to the intermediate event was expressed by

9 $$ P\left(s=1|\mathbf{x}\right)=\pi \left(\mathbf{x}\right)=\frac{1}{1+\exp \left(-\left({\gamma}_0+{\boldsymbol{\upgamma}}^T\mathbf{x}\right)\right)} $$

where the value *γ*_0_ was determined based upon iterative computation [39] to obtain a desired susceptible proportion. All covariate coefficients were set to be 1 (i.e., **γ** = **1**) for simplicity. Then, the susceptibility of each patient was generated from a Bernoulli distribution with the probability of *P*(*s*_*i*_ = 1| **x**_*i*_) as calculated in eq. (9).

#### Time to the intermediate event

For insusceptible subpopulation, the intermediate event time *T*_*e*_ was set to be a missing value because the event could never be observed. For the susceptible, the time to the intermediate event was generated from a Cox model with the baseline hazard of a Weibull distribution (*λ*_*e*_, *ν*_*e*_) and the covariate **x**. It was expressed as

10 $$ {T}_e={\left[\frac{-\log (u)}{\lambda_e\exp \left({{\boldsymbol{\upbeta}}_{\mathbf{e}}}^T\mathbf{x}\right)}\right]}^{1/{v}_e} $$

where *u* was the random number from the uniform distribution in (0,1) and **β**_**e**_ was the coefficient vector with the element being the logarithm of the hazard ratio (*HR*) for each covariate. For illustration, we set *β*_*e*_ = 1.2 for dichotomous covariates, *β*_*e*_ = 0.12 for the covariate following uniform distribution in (0,10) and *β*_*e*_ = 0.24 for the covariate following uniform distribution in (0,5) so that the covariate effects were of the same level, i.e., the multiplicative effect of each covariate on the baseline hazard (exp(*β*_*e*_*x*)) ranged from 1 to exp(1.2). *ν*_*e*_ was the shape parameter of the Weibull distribution with *ν*_*e*_ = 1 as an exponential distribution representing the intermediate event rate was constant over time. A value of *ν*_*e*_ > 1 indicated that the intermediate event rate increasing over time and a value of 0 < *ν*_*e*_ < 1 indicated the rate decreasing over time. We set *ν*_*e*_ at 0.8, 1.0, and 1.2 to cover the above three scenarios. The scale parameter of the Weibull distribution (*λ*_*e*_) was set to make sure that approximately 30–40% of the susceptible patients have experienced the intermediate event within 2 months. Accordingly, we had *λ*_*e*_ = 0.005,   0.0115, and 0.0015 for the covariate scenarios (i)-(iii), respectively.

#### Time to the outcome

We assumed that the time to the outcome for the susceptible and insusceptible subpopulations followed the same baseline Weibull distribution with parameters (*λ*_*o*_, *ν*_*o*_) but was differently affected by covariates. For illustration, we assumed that the covariates had less effect on the hazard of outcome for the insusceptible, i.e., **β**_**o,insus**_ = *ω***β**_**o,sus**_, 0 < *ω* < 1, where **β**_**o,insus**_ and **β**_**o,sus**_ were the coefficient vectors of covariate vector **x** on the hazard of the outcome for the insusceptible and susceptible subpopulations, respectively, and *ω* was the covariate effect ratio. Besides, to ensure that about half of the susceptible patients have experienced the intermediate event before the censoring of the outcome, we assumed the covariates had the same effect on the hazards of both the intermediate event and the outcome for the susceptible subpopulation. Therefore, we had *λ*_*o*_ = *λ*_*e*_ = *λ*, *ν*_*o*_ = *ν*_*e*_ = *ν*, and **β**_**o,insus**_/*ω* = **β**_**o,sus**_ = **β**_**e**_ = **β**. Accordingly, the time to the outcome for the insusceptible subpopulation was generated by

11 $$ {T}_{o, insus}={\left[\frac{-\log (u)}{\lambda_o\exp \left({{\boldsymbol{\upbeta}}_{\mathbf{o},\mathbf{insus}}}^T\mathbf{x}\right)}\right]}^{1/{v}_o}={\left[\frac{-\log (u)}{\lambda \exp \left(\omega {\boldsymbol{\upbeta}}^T\mathbf{x}\right)}\right]}^{1/v}. $$

For the susceptible subpopulation, a time-varying variable denoting the intermediate event status was added in the hazard function which was expressed as

12 $$ h\left({t}_{o, sus}\right)={\lambda}_o{\nu}_o{t_{o, sus}}^{\nu_o-1}\exp \left({{\boldsymbol{\upbeta}}_{\mathbf{o},\mathbf{sus}}}^T\mathbf{x}+{\beta}_zz\left({t}_{o, sus}\right)\right)=\lambda \nu {t_{o, sus}}^{\nu -1}\exp \left({\boldsymbol{\upbeta}}^T\mathbf{x}+{\beta}_zz\left({t}_{o, sus}\right)\right). $$

According to Austin’s work [40], we generated the time to the outcome for the susceptible subpopulation as follows:

13 $$ {T}_{o, sus}=\left\{\begin{array}{l}{\left[\frac{-\log (u)}{\lambda \exp \left({\boldsymbol{\upbeta}}^T\mathbf{x}\right)}\right]}^{1/\nu },\kern0.5em if\kern0.5em -\log (u)<\lambda {T_e}^{\nu}\exp \left({\boldsymbol{\upbeta}}^T\mathbf{x}\right)\\ {}{\left[\frac{-\log (u)-\lambda \exp \left({\boldsymbol{\upbeta}}^T\mathbf{x}\right){T_e}^{\nu }}{\lambda \exp \left({\boldsymbol{\upbeta}}^T\mathbf{x}+{\beta}_z\right)}+{T_e}^{\nu}\right]}^{1/\nu },\kern0.5em if\kern0.5em -\log (u)\ge \lambda {T_e}^{\nu}\exp \left({\boldsymbol{\upbeta}}^T\mathbf{x}\right)\end{array}\right. $$

where *T*_*e*_ was the time to the intermediate event generated by eq. (10).

Suppose the maximum follow-up time was 12 months, i.e., *τ* = 12. For all patients, the observed outcome time was the smaller one between the generated outcome time and the maximum follow-up time, i.e., *t*_*o*_ = min(*T*_*o*, *sus*_ *or T*_*o*, *unsus*_, 12). For patients susceptible to the intermediate event, the observed intermediate event time was the minimum of the generated intermediate event time, outcome time, and the maximum follow-up time, i.e., *t*_*e*_ = min(*T*_*e*_, *T*_*o*, *sus*_, 12). For the insusceptible patients, *t*_*e*_ = *C*_*e*_ = min(*T*_*o*, *insus*_, 12).

Based on the simulated random number, we estimated the effect of the intermediate event on the outcome as follows. Firstly, fit the data with the mixture cure model and estimate the model parameters via the SAS macro % PSPMCM compiled by Corbière and Joly [31] (only for methods exCox2-exCox3 and LM2-LM3). Secondly, predict the susceptibility of the patients with censored intermediate event time by the LRM (only for methods exCox2 and LM2) and the RITI (only for methods exCox3 and LM3) methods. Thirdly, estimate the effect of the intermediate event on the outcome by the extended Cox regression or landmark methods. Repeat the three steps *M* times and compare the estimation performance of the eight methods by average bias (BIAS) and mean squared errors (MSE) with.

14 $$ \mathrm{BIAS}=\sum \limits_{i=1}^M\left({\hat{\beta}}_z\right)/M-{\beta}_z\kern0.5em \mathrm{and}\ \mathrm{MSE}=\sum \limits_{i=1}^M{\left({\hat{\beta}}_z-{\beta}_z\right)}^2/M $$

where ${\hat{\beta}}_z$ was the estimate of *β*_*z*_, i.e., the effect of the intermediate event on the outcome. The smaller the magnitudes of BIAS and MSE, the more accurate the estimation.

To comprehensively compare the estimation performances of methods exCox1-exCox4 and LM1-LM4 and investigate the factors that may affect the performance of the proposed new method, we carried out the simulation study in various scenarios. Specifically, we set three covariate scenarios (as described in "Covariate vector" of this subsection), three levels for the ratio of the effect of covariates on the outcome in the insusceptible and susceptible subpopulation (i.e., *ω* = 0.5, 0.67, 0.83) and three landmark times (i.e., 1, 2, 3 months) for the landmark method. The number of simulations per scenario was *M* = 100. The sample size was set to be 2000 in each scenario to guarantee at least 40–50 outcomes were observed in the analysis dataset. SAS 9.4 (SAS Institute Inc., Cary, NC, USA) was used for simulated data generation and analysis.

### Simulation results

**Fig. 1:**
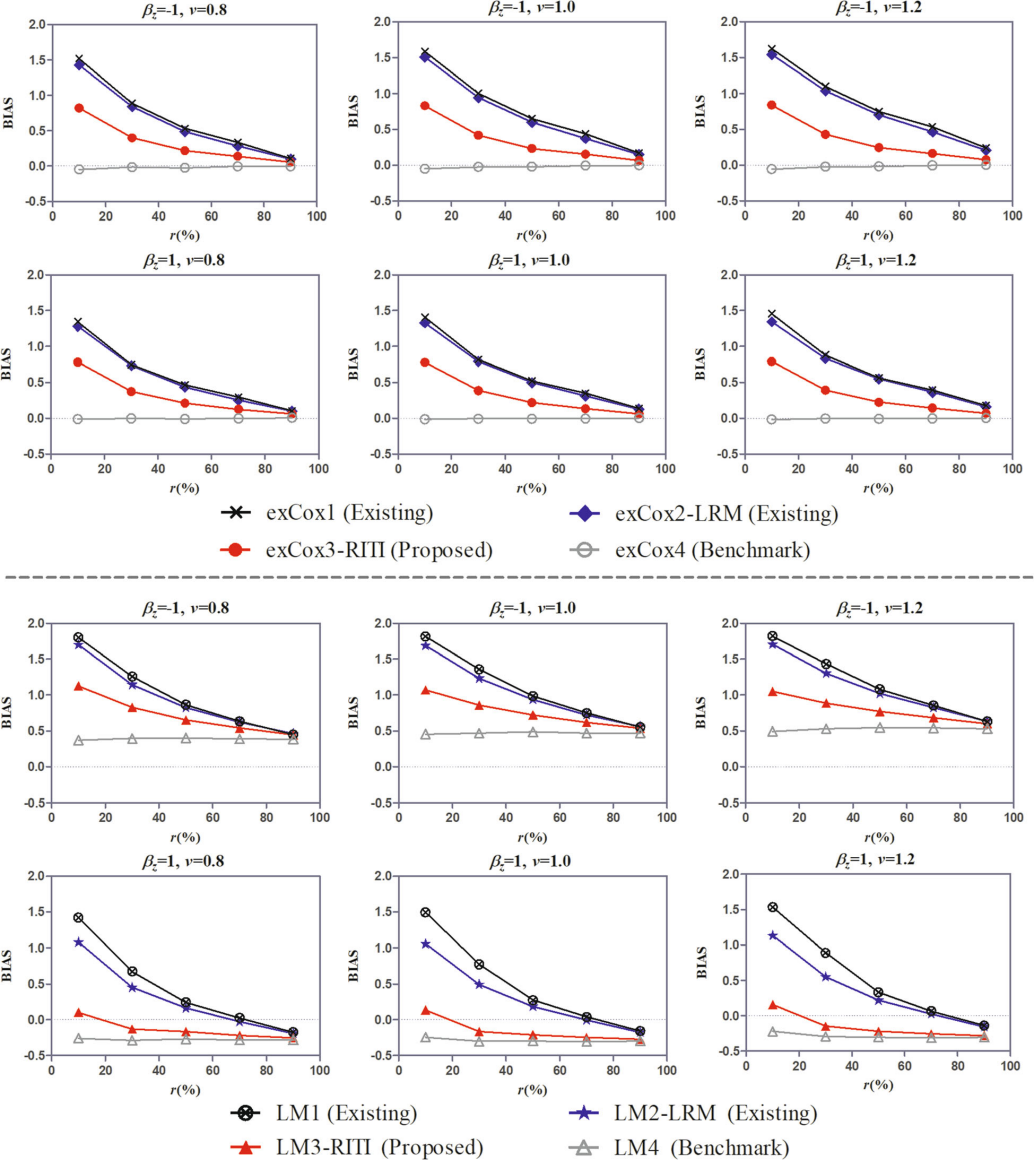
BIAS of the effect estimate under scenarios with different *β*_*z*_ and different *ν*. *β*_*z*_ is the true effect of the intermediate event on the outcome, *ν* is the shape parameter of the Weibull distribution of the intermediate event time and the outcome time, and *r*(%) is the proportion of the susceptible in the study population

**Fig. 2:**
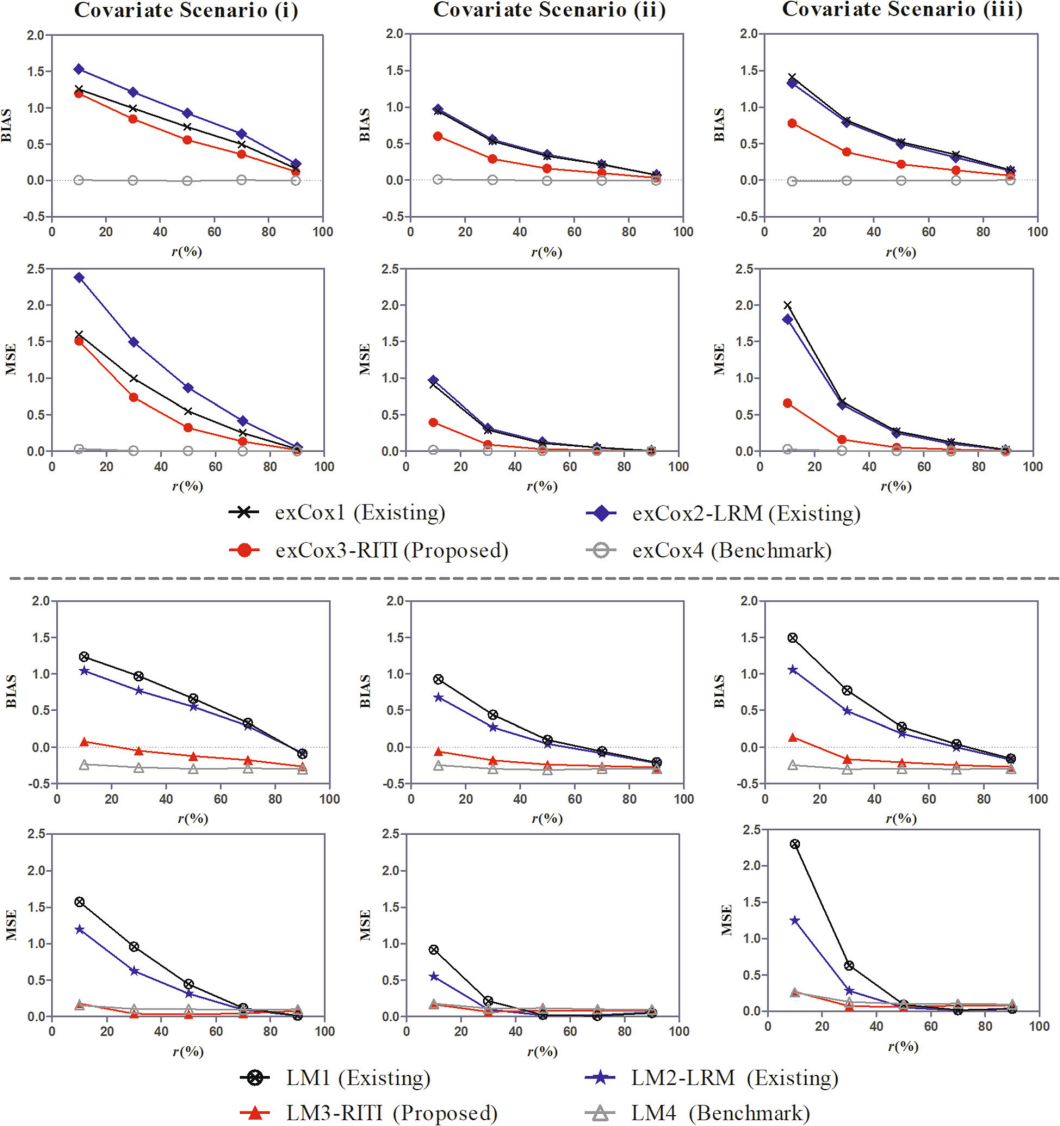
BIAS and MSE of the effect estimate under covariate scenarios (i)-(iii)

**Fig. 3:**
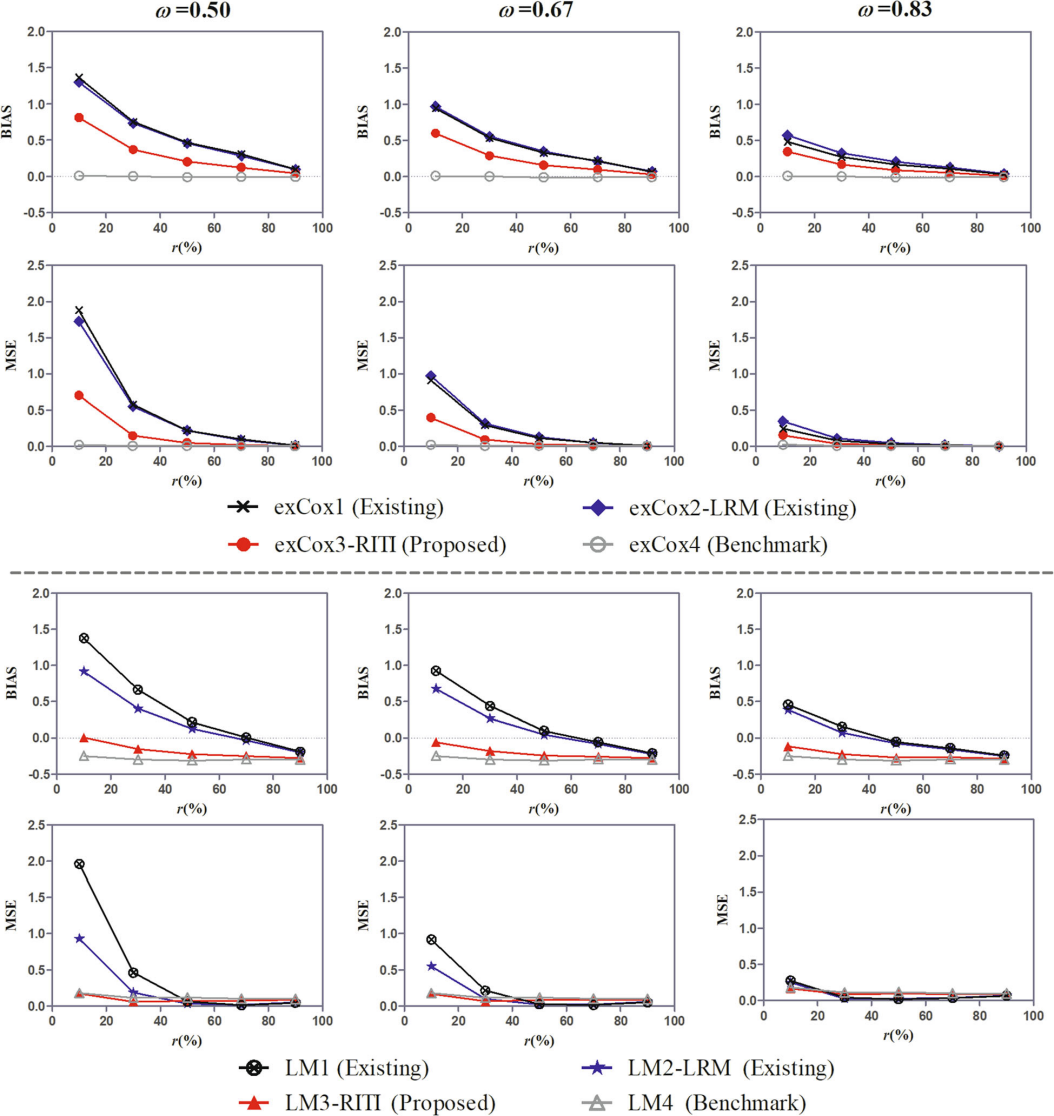
BIAS and MSE of the effect estimate under scenarios of different covariate effect ratios (*ω*)

**Fig. 4:**
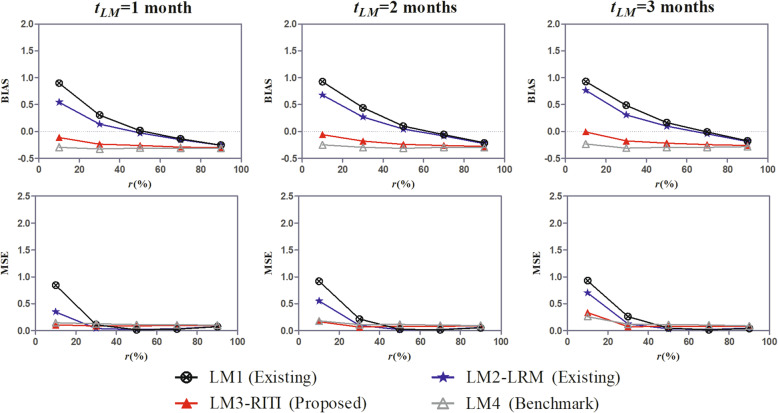
BIAS and MSE of the effect estimate under scenarios of different landmark times (*t*_*LM*_)

We display the simulation results of methods exCox1-exCox4 and LM1-LM4 separately since they belong to two series of methods. For methods with the prefix of “exCox”, the effect was estimated by the extended Cox regression while the landmark method was adopted to estimate the effect for methods with the prefix of “LM”. In addition, to illustrate the influence of each factor, the estimation performances of these methods are investigated by varying factors one at a time while controlling the others. Specifically, Figs. 1, 2, 3, 4 in this subsection show the variation of the estimation performance with five different factors as follows.

- Different effects of the intermediate event on the outcome (*β*_*z*_) and different event rate variations, the latter was reflected by the shape parameter (*ν*) of the Weibull distribution of the intermediate event time and the outcome time (Fig. 1).
- Different covariates (scenarios (i)-(iii)) included in the study (Fig. 2).
- Different ratios of the effect of covariates on the outcome in the insusceptible and susceptible subpopulations (*ω*) (Fig. 3).
- Different landmark times (*t*_*LM*_) for the landmark method (Fig. 4).

**Fig. 5:**
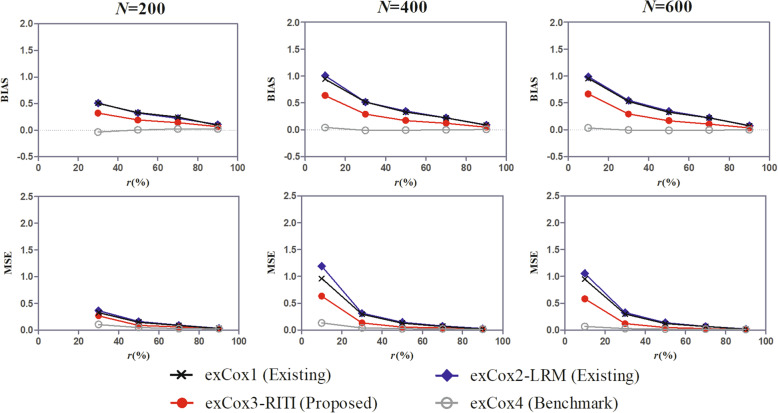
BIAS and MSE of the effect estimate in cases of small sample sizes

**Fig. 6:**
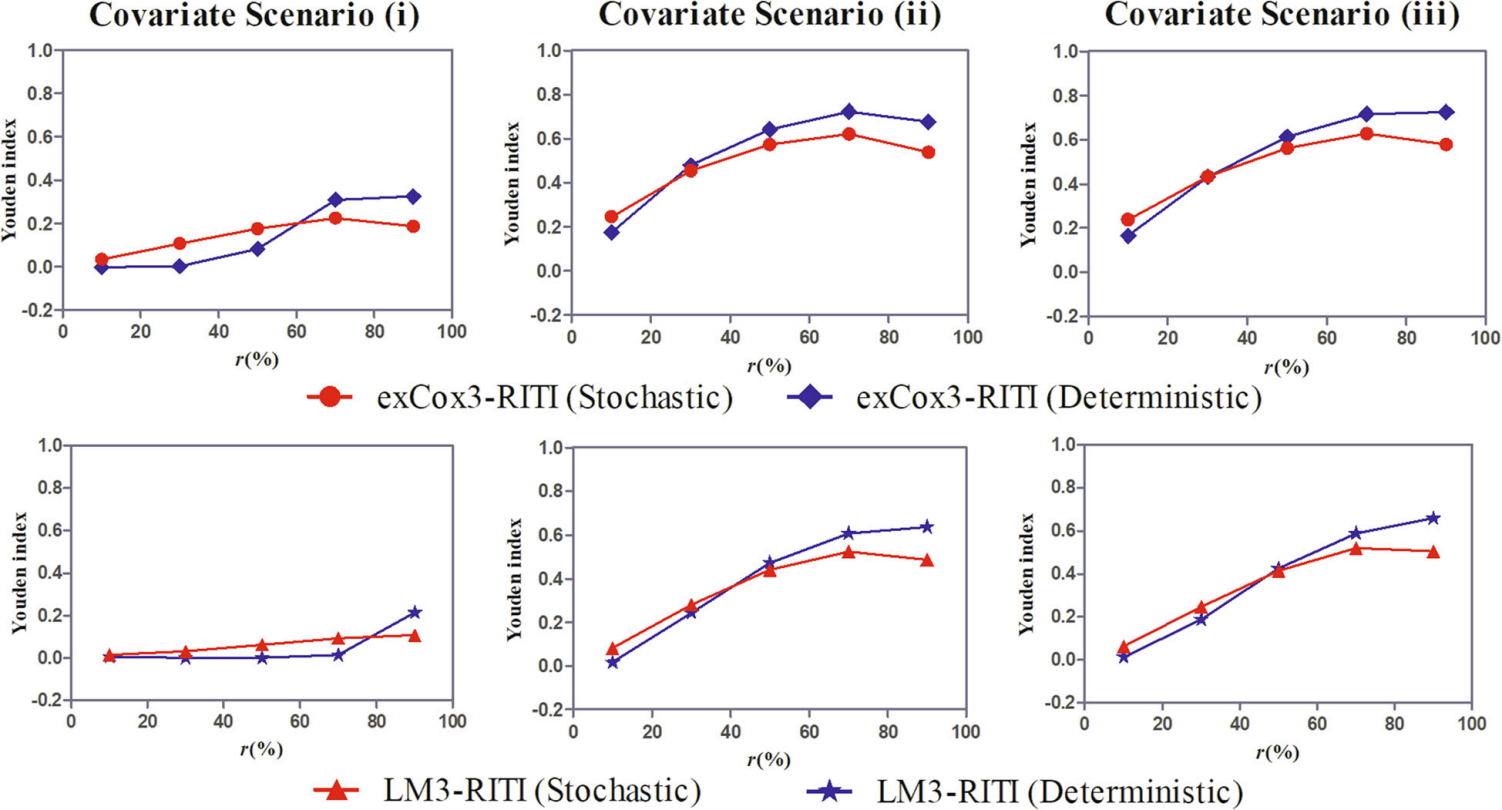
Subpopulation classification performance of the stochastic and deterministic procedures of the RITI method

**Fig. 7:**
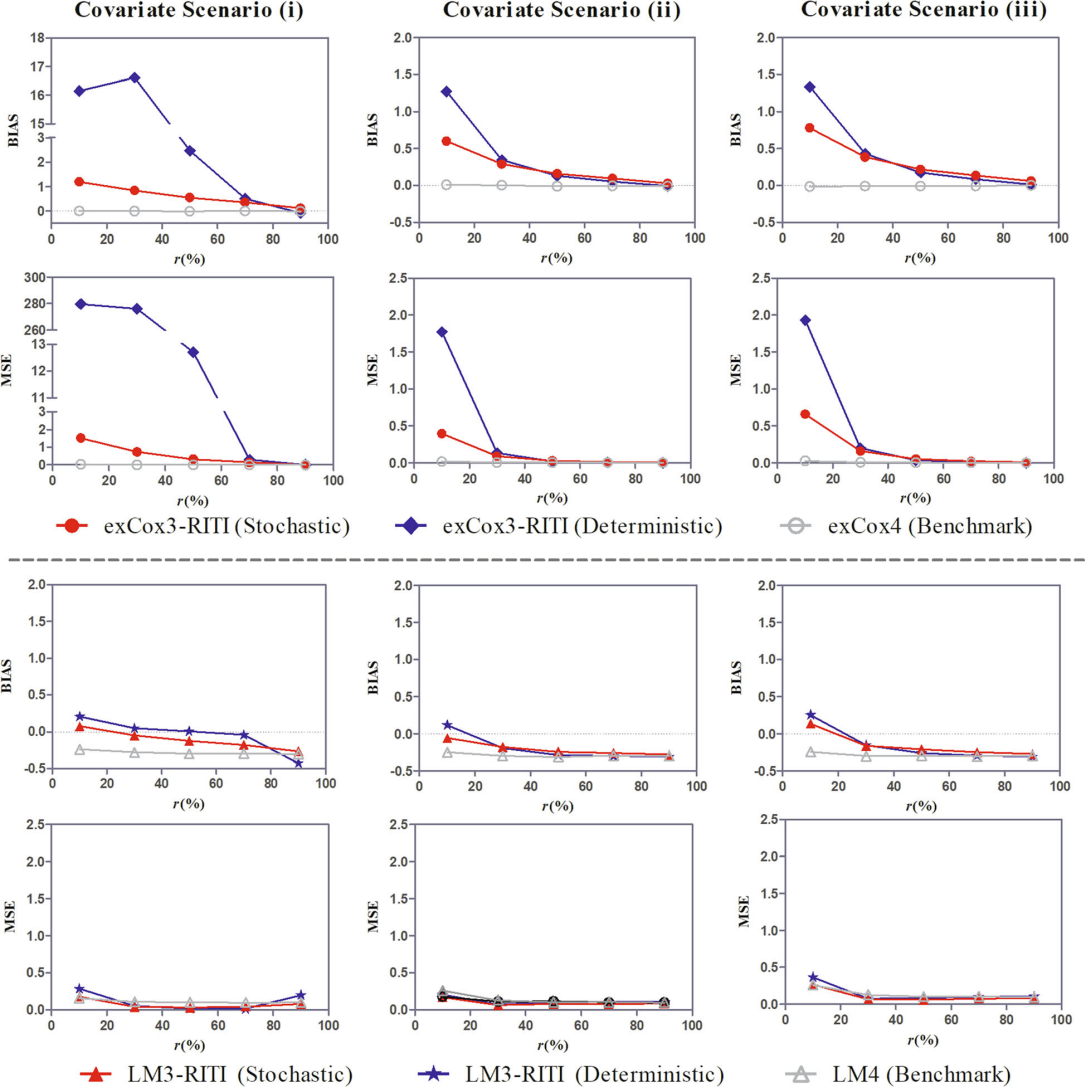
BIAS and MSE of the effect estimate using stochastic and deterministic procedures of RITI method

Besides, in Fig. 5, we examine the small sample performance of the proposed method. The comparison between the stochastic procedure and the deterministic procedure with the cutoff point of 0.5 of the proposed RITI pre-identification method is shown in Figs. 6 and 7. For each scenario, we show the BIAS and MSE of the effect estimate ${\hat{\beta}}_z$ versus the proportion of the susceptible in the study population, i.e., *r*(%).

Figure 1 shows the effect estimation performance of the proposed method, as well as other methods, under scenarios of different true effects of the intermediate event (*β*_*z*_ =  − 1 or *β*_*z*_ = 1) and different shape parameters of the Weibull distribution of the intermediate event time and the outcome time (*ν* = 0.8, 1.0, 1.2). The covariate scenario (iii) and covariate effect ratio *ω* = 0.67 were set in all scenarios. For methods LM1-LM4, the landmark time was *t*_*LM*_ = 2 months. It shows that under all scenarios, the estimation performance of the proposed new methods (exCox3 and LM3) is the closest to the performance benchmark provided by methods exCox4 and LM4. Existing methods exCox1 and LM1 bring large bias to the effect estimate. That is, the proposed new methods could remarkably reduce the estimation bias of existing methods by the susceptible subpopulation pre-identification process. However, when the LRM is employed in the susceptible pre-identification (methods exCox2 and LM2), the estimation performance is not satisfactory. The reduction of the estimation bias by methods exCox2 and LM2 is tiny. So we conclude that the RITI method performs better in identifying the susceptible than the LRM. This is because the former uses more information than the latter when identifying the susceptible. Both the incidence part and the conditional survival function of the mixture cure model are used in the RITI method while only the incidence part is used in the LRM. Additionally, we observe that when the intermediate event has a harmful effect on the outcome (*β*_*z*_ = 1), the performance gap between the proposed method LM3 and the benchmark method LM4 is smaller than that in the case of *β*_*z*_ =  − 1. In other words, the new method LM3 is more recommended to be used in the effect estimation of a harmful intermediate event.

There are two common characteristics between the proposed methods and existing methods. (a) Compared to the benchmark methods exCox4 and LM4, methods exCox1-exCox3 and LM1-LM3 provide numerically larger point estimates of the effect (i.e., ${\hat{\beta}}_z$), which is characterized by the larger BIAS in Fig. 1. Because in the setting of this paper, the inclusion of the insusceptible subpopulation reduces the hazard of the outcome in the event-free group. In the case of *β*_*z*_ =  − 1 (*β*_*z*_ = 1), i.e., the intermediate event has a protective (harmful) effect on the outcome, the decrease of the hazard of the outcome in the event-free group leads to the decrease (increase) of the hazard gap between the event group and the event-free group, leading to the underestimation (overestimation) of the protective (harmful) effect further. In both cases, the effect estimate (${\hat{\beta}}_z$), as well as the BIAS, is numerically larger. (b) When the proportion of the susceptible in the study population increases, the estimation biases of methods exCox1-exCox3 and LM1-LM3 gradually decrease to the level of methods exCox4 and LM4, respectively, since the impact of the insusceptible population is fading away. In addition, we observe that the estimation bias of method exCox4 is close to zero in all scenarios, which is not true for method LM4. This demonstrates that without the mix of the insusceptible subpopulation, the extended Cox regression method could provide a more accurate effect estimate than the landmark method, which has also been reported in the literature [4].

When it comes to the estimation performance of the proposed method and other methods under scenarios of different *β*_*z*_ and *ν*, we find that the estimation biases of methods exCox1-exCox4 are similar in scenarios of *β*_*z*_ =  − 1 and *β*_*z*_ = 1, which is not true for methods LM1-LM4. Specifically, the estimation biases of methods LM1-LM4 are consistently positive when *β*_*z*_ =  − 1. In the case of *β*_*z*_ = 1, the estimation bias of the benchmark method LM4 is always negative, while the estimation biases of methods LM1-LM3 go from positive to negative, eventually, approaching to the bias of method LM4, as the proportion of the susceptible increases. From the estimation bias of method LM4, we conclude that without the mix of the insusceptible, the landmark method underestimates the effect, whether protective or harmful, of the intermediate event. This is because the landmark method groups the patients based on the intermediate event status at the landmark time, which leads to misclassification to some extent. The misclassification reduces the gap between the event group and the event-free group and decreases the effect difference between groups. The shape parameter of the Weibull distribution (*ν*) has a small impact on the estimation performances of methods exCox1-exCox4 and LM1-LM4. With the increase of *ν*, the estimation biases of methods LM1-LM4 increase, but to a very small extent. For example, in the case of *β*_*z*_ =  − 1 and *r* = 10%, the estimation bias of method LM4 increases from 0.37, 0.45 to 0.49 with *ν* changing from 0.8, 1.0 to 1.2. Because with the increase of *ν*, the intermediate event occurs later, the landmark method would produce more misclassification and lead to larger estimation bias. For methods exCox1-exCox3, the estimation biases also increase with *ν*. For example, in the case of *β*_*z*_ =  − 1 and *r* = 10%, the estimation bias of method exCox3 increases from 0.82, 0. 83 to 0.84 with *ν* changing from 0.8, 1.0 to 1.2. This is because more susceptible patients could not experience the intermediate event due to the later occurrence of the event, the susceptibilities of more patients are left to be predicted, which would lead to larger bias.

From Fig. 1, we observe that the effect of the intermediate event (*β*_*z*_) and the shape parameter of the Weibull distribution (*ν*) have a small impact on the estimation performance difference among methods exCox1-exCox4 and LM1-LM4. Therefore, in the following figures, the results under the scenarios of *β*_*z*_ =  − 1 and *ν* = 0.8, 1.2 are not displayed for visual clarity and space-saving.

Figure 2 shows the estimation performance of methods exCox1-exCox4 and LM1-LM4 under different covariate scenarios. The covariate effect ratio was *ω* = 0.67 in all scenarios. For methods LM1-LM4, the landmark time was *t*_*LM*_ = 2 months. As described in the part of “Simulation Setting”, there are four categorical covariates in covariate scenario (i). Two of them are substituted by continuous covariates in covariate scenario (ii). In covariate scenario (iii), two more continuous covariates are added compared to covariate scenario (i) while two more categorical covariates are added compared to covariate scenario (ii). The results show that the proposed method reduces the estimation bias of existing methods in all scenarios, though the magnitude of the bias reduction varies with the covariate scenarios. Compared to covariate scenario (i), the performance superiority of the proposed method over existing methods is greater in covariate scenario (ii). This is because the continuous covariates contain more information than categorical covariates, the susceptible pre-identification is more accurate in covariate scenario (ii). It is observed that the estimation BIAS and MSE of method exCox2 are larger than those of method exCox1 in covariate scenario (i). A reason is that the LRM in method exCox2 classifies more insusceptible patients into the susceptible, which leads to a larger insusceptible proportion in the predicted susceptible subpopulation than in the entire population. Therefore, the LRM for the insusceptible pre-identification is not reliable.

When comparing the results in covariate scenarios (ii) and (iii), we find the performance difference among methods exCox1-exCox4 and LM1-LM4 are similar but the BIAS and MSE are larger in covariate scenario (iii). The reason is that with the same sample size, the increase of covariates decreases the statistical power. Compared to covariate scenarios (ii), the two categorical covariates added in covariate scenario (iii) do not improve the accuracy of the susceptible pre-identification. On the contrary, the proposed method exCox3 performs better in covariate scenario (iii) than in covariate scenario (i). It is also true for method LM3 when the susceptible proportion is not too small (*r* ≥ 30%) since the two continuous covariates added in scenario (iii) increases the accuracy of the susceptible pre-identification. However, when *r* = 10%, the method LM3 performs a little better in covariate scenario (i) than in covariate scenario (iii), which could be attributed to the relatively small sample size in covariate scenario (iii).

To sum up, the proposed new method could reduce the bias caused by the mix of the insusceptible subpopulation by pre-identifying the susceptible. More covariates, especially continuous covariates, could increase the effect of the susceptible pre-identification process. However, both the covariate number and the sample size impact the estimation performance of the proposed method. The decrease of the estimation bias caused by the increase of the covariate number may be neutralized by the increase of the estimation bias caused by the relative decrease of the sample size. Therefore, rather than increasing the covariate number, more discriminative covariates should be included in the insusceptible pre-identification process.

Figure 3 shows the BIAS and MSE of the effect estimate of methods exCox1-exCox4 and LM1-LM4 under the covariate scenario (ii) with different covariate effect ratios. For methods LM1-LM4, the landmark time was *t*_*LM*_ = 2 months. It is observed that the BIAS and MSE of the effect estimate of methods exCox1-exCox3 and LM1-LM3 decrease with the increase of the covariate effect ratio (*ω*). This is because when *ω* being closer to one, the heterogeneity between the insusceptible and susceptible subpopulations decreases, and the impact of including the insusceptible patients in analysis decreases. In all considered scenarios, the performance of the proposed method is still better than that of the existing methods and is closer to the performance benchmark. The results in Fig. 3 also confirm the robustness of the proposed method to the covariate effect ratio.

The BIAS and MSE of the effect estimate of methods LM1-LM4 under covariate scenario (ii), covariate effect ratio *ω* = 0.67, and different landmark times are shown in Fig. 4. It shows that the superiority of the proposed method over existing methods is consistent in different landmark times. Nevertheless, the landmark time influences the estimation performance of all methods, especially in the case of a small susceptible rate. Specifically, the BIAS of the effect estimate of the method LM4 is closer to zero at later landmark times, though to a small extent. It is on account of the less misclassification at later landmark times. The estimation performances of methods LM1-LM3 are closer to the performance benchmark provided by the method LM4 at early landmark times. In addition, for methods LM1-LM4, the MSE of the effect estimate increases with the landmark time when the proportion of the susceptible is small. This is because more data are discarded from the analysis at later landmark times, which leads to smaller sample sizes and potential loss of power.

To examine the small sample performance of the proposed method, we conducted a simulation study with sample sizes of 200, 400, and 600. Considering that patients with outcome occurred before the landmark time are excluded from the analysis for the landmark method, which makes the sample size smaller, we used extended Cox regression to estimate the effect among the identified susceptible subpopulation. The true effect of the intermediate event *β*_*z*_ = 1, shape parameter of the Weibull distribution *ν* = 1.0, covariate effect ratio *ω* = 0.67, and the covariate scenario (ii) were set in the simulation. The results are shown in Fig. 5.

It shows that the superiority of the proposed method over existing methods maintains with the small sample size. The effect estimate of the proposed method exCox3 is more accurate than that of the methods exCox1-exCox2. Besides, the sample size has little effect on the BIAS of the effect estimate. While the MSE of the effect estimate decreases slightly with the sample size, especially in the case of a small susceptible rate (*r* ≤ 30%), which could be attributed to the increased power with larger sample sizes.

In the case of *N* = 200, we have considered only the scenarios of the susceptible rate *r* ≥ 30% instead of *r* ≥ 10%. Because the number of susceptible patients is about 20 and the number of the observed intermediate event might fall into the single digits when *r* = 10%, which is unlikely to provide sound conclusions in reality.

For patients without the intermediate event, the proposed RITI method determines whether the patient is susceptible to the intermediate event according to whether the residual intermediate event time could be calculated. In essence, the susceptibility is determined by a Bernoulli distribution with the probability of $\frac{1-\pi \left(\mathbf{x}\right)}{1-\pi \left(\mathbf{x}\right)+\pi \left(\mathbf{x}\right)S\left({C}_e|s=1,\mathbf{x}\right)}$, which we call the stochastic procedure. In this sense, an alternative is to take 0.5 as the cutoff point and classify a patient as insusceptible if $\frac{1-\pi \left({\mathbf{x}}_i\right)}{1-\pi \left({\mathbf{x}}_i\right)+\pi \left({\mathbf{x}}_i\right)S\left({C}_{ei}|s=1,{\mathbf{x}}_i\right)}>0.5$, which we call the deterministic procedure. In Fig. 6 and Fig. 7, we compare the stochastic procedure and the deterministic procedure with 0.5 as the cutoff point of the proposed RITI classification method in terms of the subpopulation classification performance and the effect estimation accuracy, respectively. The simulation was conducted under covariate scenarios (i)-(iii) since the covariates could influence the classification performance and the effect estimation as illustrated in Fig. 2. We set the true effect *β*_*z*_ = 1, the shape parameter of the Weibull distribution *ν* = 1.0, and the covariate effect ratio *ω* = 0.67 in all scenarios. The landmark time was *t*_*LM*_ = 2 months when estimating the effect with the landmark method.

In Fig. 6, the subpopulation classification performance is evaluated by the Youden index [41], which is calculated by adding the rate that the susceptible are correctly classified as susceptible to the rate that the insusceptible are correctly classified as insusceptible, then subtracting one from that value. The larger the Youden index, the more reliable the classification. Figure 6 shows that the stochastic and deterministic procedures have comparable classification performances. That is, the classification is not very sensitive to the stochastic cutoff. When the susceptible rate is small, the stochastic procedure produces more accurate classification than the deterministic procedure while the deterministic procedure outperforms the stochastic procedure when the susceptible rate is large.

By in-depth exploration, we find that when the susceptible rate in the population is small, the value of $\frac{1-\pi \left(\mathbf{x}\right)}{1-\pi \left(\mathbf{x}\right)+\pi \left(\mathbf{x}\right)S\left({C}_e|s=1,\mathbf{x}\right)}$ for susceptible patients without the intermediate event exhibits a negative skew distribution. Many susceptible patients with the censored intermediate event are incorrectly categorized into the insusceptible group based on the 0.5 cutoff point of the deterministic procedure. Particularly, under the covariate scenario (i), nearly all the susceptible patients with the censored intermediate event have $\frac{1-\pi \left(\mathbf{x}\right)}{1-\pi \left(\mathbf{x}\right)+\pi \left(\mathbf{x}\right)S\left({C}_e|s=1,\mathbf{x}\right)}>0.5$ in the case of *r* ≤ 30%, the deterministic procedure with 0.5 as the cutoff point has little effect in identifying the susceptible. Only the susceptible patients with the observed intermediate event are included in the analysis. In this case, the stochastic procedure identifies more susceptible patients since there is a chance for patients with $\frac{1-\pi \left(\mathbf{x}\right)}{1-\pi \left(\mathbf{x}\right)+\pi \left(\mathbf{x}\right)S\left({C}_e|s=1,\mathbf{x}\right)}>0.5$ to be classified as susceptible. On the contrary, with the increase of the susceptible rate, the value of $\frac{1-\pi \left(\mathbf{x}\right)}{1-\pi \left(\mathbf{x}\right)+\pi \left(\mathbf{x}\right)S\left({C}_e|s=1,\mathbf{x}\right)}$ for susceptible patients without the intermediate event gradually exhibits positive skew distributions. Most of the susceptible patients with the censored intermediate event are categorized into the susceptible group based on the 0.5 cutoff point of the deterministic procedure.

Figure 7 shows that the effect estimate by the stochastic procedure of the proposed RITI classification method is more accurate than that based on the deterministic procedure when the susceptible rate in the study population is small. With the increase of the susceptible rate, the estimation performances based on the two procedures are comparable while the deterministic procedure shows a little superiority over the stochastic procedure.

Combining Fig. 6 and Fig. 7, we find that the more accurate classification of the stochastic procedure in small susceptible rate scenarios provides much more accurate effect estimates, while the classification superiority of the deterministic procedure in large susceptible rate scenarios has little help in improving the accuracy of the effect estimate. The possible reason is that when the susceptible rate is small, the sample size of the susceptible subpopulation is small, and then the effect estimate is more sensitive to the classification accuracy. From the perspective of the purpose of the study, i.e., obtaining a more accurate effect estimate, the deterministic procedure with 0.5 as the cutoff point may not be appropriate for all cases, while the stochastic procedure of the RITI classification method is widely applicable and has relatively robust and well performance.

**Fig. 8:**
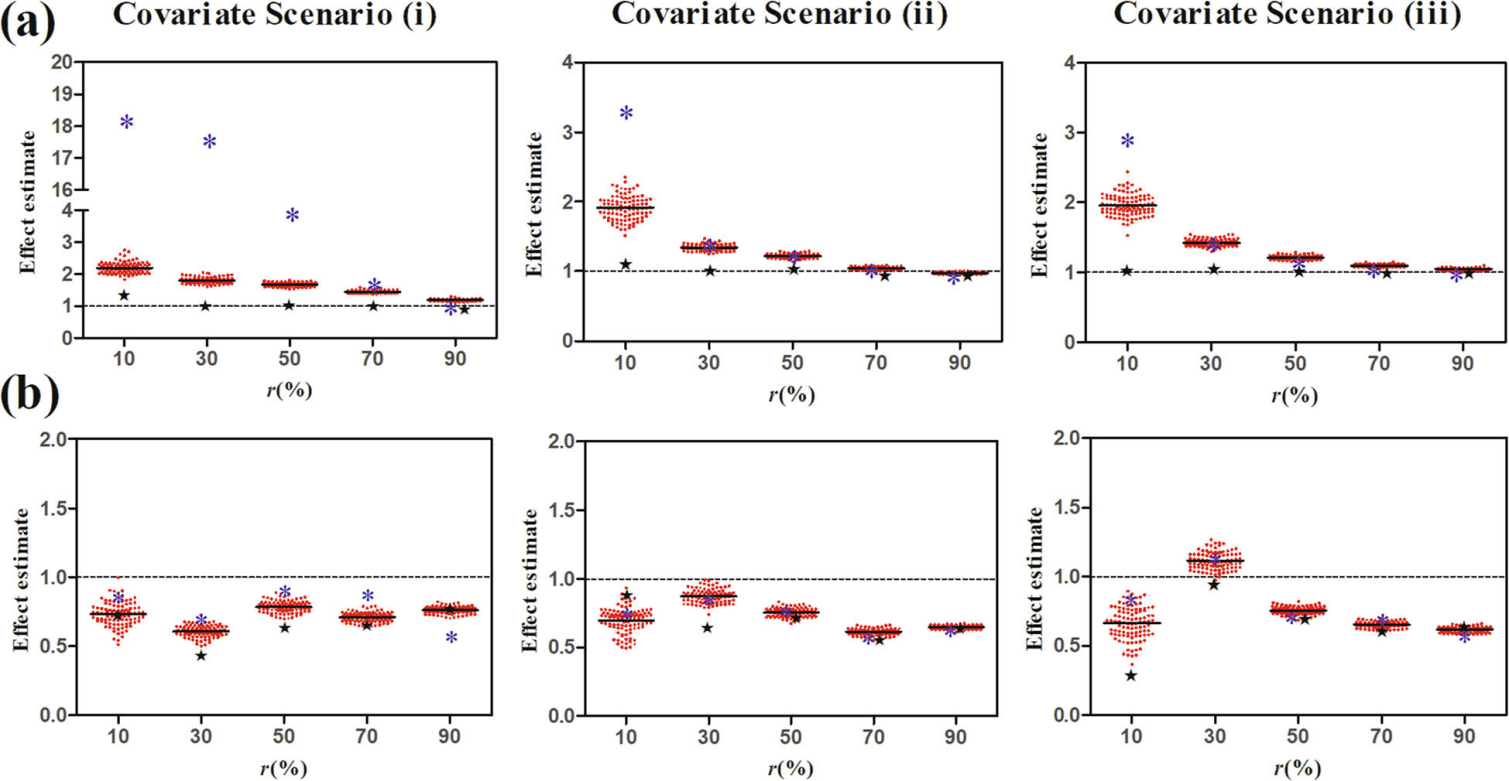
Scatter plot of effect estimates with the stochastic procedure of RITI based on one dataset. Based on one simulated dataset generated in the setting of Fig. 7, 100 classification datasets and the corresponding effect estimates (denoted by red dot) were obtained with the stochastic procedure of the RITI classification method. The short black solid lines represent the mean value of the 100 effect estimates. The effect estimates with the deterministic procedure of the RITI classification method (denoted by *) and the benchmark method (denoted by ★) based on the same dataset were also illustrated for comparison purposes. Methods used on the top panel (**a**): exCox3-RITI (Stochastic), exCox3-RITI (Deterministic), and exCox4 (Benchmark); the bottom panel (**b**): LM3-RITI (Stochastic), LM3-RITI (Deterministic), and LM4 (Benchmark)

For the stochastic procedure of the RITI classification method, a potential concern might be the reproducibility of the effect estimate as different analysts could make different classifications due to the stochastic cutoff. To investigate the possible variation of the effect estimate, we generated 100 classification datasets by identifying the susceptible patients with the stochastic procedure based on one simulated dataset and obtained the effect estimates separately. Based on the same simulated dataset, the effect estimate with the benchmark methods (exCox4 and LM4) and the effect estimate with the deterministic procedure of the RITI classification method were also calculated. The scatter plot of the 100 effect estimates with the stochastic procedure, the benchmark estimate, as well as the corresponding effect estimate with the deterministic procedure are shown in Fig. 8. We find that the variation of the effect estimate based on the stochastic procedure of the RITI classification method decreases with the susceptible rate. When the susceptible rate is not too small (i.e., *r* ≥ 30%), the estimates are close to each other. That is, the estimate based on the stochastic procedure changes little with analysts. In the case of *r* = 10%, the variation of the estimate is not negligible. However, when *r* = 10%, the average estimate with the stochastic procedure is closer or similarly close to the benchmark estimate in comparison with that of the deterministic procedure. Especially, for method exCox3, all the estimates based on the stochastic procedure are much closer to the benchmark estimate when *r* = 10% (shown in Fig. 8(a)). Considering the estimate based on small sample sizes is not robust enough for the overall inference, the slight variation of the estimate in the case of *r* = 10% is still acceptable. Therefore, the reproducibility issue of the stochastic procedure has a negligible impact on the conclusion of the analysis.

### Case study

Mycosis fungoides (MF) is a common cutaneous T cell lymphomas (CTCLs). Advanced-stage patients have dismal prognoses, with a life expectancy fewer than 4 years. However, more than 80% of patients at early-stage (IA or IB) will have an indolent lifelong course free of disease progression [42]. The susceptible patients would experience the disease progression or death within 10 years. The tumor clone frequency (TCF) in lesional skin (> 25%), disease stage (IB versus IA), and age (> 60 years) are sensitive factors to predict which patient might progress and the progress/death time [43]. In this instance, the inclusion of the insusceptible patients may lead to biased effect estimation of the disease progression on survival. According to de Masson’s work [43], we simulated the TCF, disease stage, age, susceptibility to progress, progress time for susceptible patients, and death time for all MF patients in a dataset. Then the proposed method (exCox3-RITI) and two existing methods (exCox1 and exCox2-LRM) were applied to estimate the effect of the disease progression on survival. The results are shown in Table 3. Parameter setting and considerations for the simulated dataset, as well as the SAS codes, are in Additional file 1.

**Table 3:** The effect of the disease progression on the survival of MF patients

Variables	Real effect *β*_*z*_	exCox1 (Existing)		exCox2-LRM (Existing)		exCox3-RITI (Proposed)	
		${\hat{\beta}}_z$	*P* value	${\hat{\beta}}_z$	*P* value	${\hat{\beta}}_z$	*P* value
TCF	1.6	1.006	< 0.001	1.335	< 0.001	2.286	< 0.001
Stage	0.9	0.431	< 0.001	0.841	< 0.001	1.258	< 0.001
Age	0.7	1.356	< 0.001	1.115	< 0.001	1.067	< 0.001
Progress	2.0	3.139	< 0.001	3.575	< 0.001	2.724	< 0.001

As shown in Table 3, the proposed method (exCox3-RITI) provides a more accurate estimate of the effect of “progress” on survival than the existing two methods (exCox1 and exCox2-LRM), which is in line with the simulation results. The effect of the disease progress estimated by exCox3-RITI is more close to the real value, with a small bias. Ignoring the insusceptible patients and including all patients in the analysis (exCox1) bring large bias to the effect estimation. Susceptible patient pre-identification via the LRM is not reliable and does not improve the effect estimation accuracy of the disease progress (exCox2-LRM).

## Discussion

In this paper, we aim to estimate the effect of the time-varying intermediate event on the outcome when there is an insusceptible fraction to the intermediate event in the study population. Existing methods neglect the existence of the insusceptible subpopulation, which brings bias to the effect estimate. An improved new method is proposed, in which the susceptible identification is performed firstly using the RITI method. Then the effect of the intermediate event on the outcome is estimated via the extended Cox regression and landmark methods based on the predicted susceptible population.

The simulation study in various scenarios demonstrates that the proposed effect estimation method based on the susceptible subpopulation pre-identification dramatically reduces the estimation bias of existing methods. Based on the real susceptible subpopulation, the extended Cox regression could provide an unbiased estimate of the effect, while the landmark method underestimates the effect, whether protective or harmful, of the intermediate event, which is consistent with the results in Mi’s research [4]. When the insusceptible subpopulation is included in the analysis of the extended Cox regression and landmark methods, the effect estimate is biased and the bias increases with the proportion of the insusceptible. The susceptible subpopulation pre-identification in the proposed method helps to reduce the impact of the insusceptible subpopulation and improve the effect estimation accuracy significantly.

When it comes to the method for the susceptible subpopulation pre-identification, the proposed RITI method shows great superiority to the existing classification method, i.e., the LRM method. The estimation bias of the proposed method is smaller than that of the method where the LRM is used to identify the susceptible. Particularly, when the intermediate event has a harmful effect on the outcome and the effect is estimated via the landmark method, the result based on the susceptible subpopulation identified by the LRM is contrary to reality. So the RITI method is more reliable than the LRM method. That is because the RITI method takes advantage of both the incidence and time information of the intermediate event while the LRM only uses the incidence information of the intermediate event. By exploiting more information, the RITI method distinguishes the insusceptible and the susceptible more accurately. In addition, the comparison between the stochastic procedure and the deterministic procedure with 0.5 as the cutoff point of the RITI classification method illustrates that the stochastic procedure is widely applicable with relatively robust and well performance. Despite the reproducibility issue, the impact is negligible on the conclusion of the analysis. Therefore, the stochastic procedure of the RITI method is more recommended. In cases that reproducibility is seriously concerned, the deterministic procedure could also be adopted if the susceptible rate is large. Despite the much-reduced bias of the effect estimate, the performance of the proposed method is not perfect. There are still insusceptible patients in the identified susceptible subpopulation, which leads to a gap between the effect estimate to the real value.

Covariates used in identifying the susceptible subpopulation have a major influence on the performance of the proposed method because they can affect the identification accuracy directly. The estimation performance of the proposed method is closer to the performance benchmark when there are more covariates, especially continuous covariates, because of the more accurate susceptible subpopulation pre-identification. However, the estimation bias of the new method is jointly affected by covariates and the sample size. Under the same sample size, more covariates could increase the identification accuracy, but at the same time would lead to increased bias because of the decreased statistical power. In cases with the same number of covariates, the continuous covariates are more helpful in distinguishing the insusceptible and the susceptible compared to the categorical covariates. Therefore, continuous covariates with high discrimination ability should be included to make the proposed method perform better. Besides, the heterogeneity of the effect of covariates on the outcome in insusceptible and susceptible subpopulations has an impact on the effect estimation performance of all considered methods. The estimation bias is larger when the effect heterogeneity increases since the impact of including the insusceptible in the analysis increases. For methods that estimate the effect via the landmark time, the estimation bias is smaller at later landmark time. Because less misclassification occurs at the later landmark time. Both the effect heterogeneity of the covariates and the landmark time have little impact on the performance superiority of the proposed method over the existing methods.

The improved method we proposed in this paper hopes to perform the effect estimation in the right population to reduce the bias caused by the mix of the insusceptible subpopulation. The susceptible subpopulation pre-identification is the core idea we proposed and the RITI method based on the fitted mixture cure model is the tool we used to achieve the pre-identification. The simulation study confirms the superiority of the improved method. However, the estimation bias could be reduced but could not be erased by the proposed method since the RITI classification method could not separate heterogeneous subgroups completely. Other methods such as the more flexible nonparametric cure models [21] and latent class models [44, 45] could be resorted to improve the pre-identification accuracy. The extension of the proposed method with time-dependent covariates and more flexible models will be pursued in our future research.

## Conclusions

Based on the pre-identification of the susceptible, the proposed new method could improve the effect estimation accuracy of the intermediate event on the outcome when there is an insusceptible fraction to the intermediate event in the study population.

## Supplementary Information

**Additional file 1.** Contains the parameter setting and considerations, as well as the SAS codes, for the simulated mycosis fungoides dataset used in the case study.

## Data Availability

SAS codes for the simulated mycosis fungoides dataset used in the case study are publicly available in Additional file 1.

## Abbreviations

aGVHD: Acute graft-versus-host disease
PDF: Probability density function
EM: Expectation-maximization
LRM: Logistic regression model
RITI: Residual intermediate-event time imputation
LM: Landmark method
HR: Hazard ratio
MSE: Mean squared errors
MF: Mycosis fungoides
CTCLs: Cutaneous T cell lymphomas
TCF: Tumor clone frequency
