# Supplementary material for: An improved method for the effect estimation of the intermediate event on the outcome based on the susceptible pre-identification
Source: BMC Med Res Methodol. 2021 Sep 21;21:192. doi: 10.1186/s12874-021-01378-8 (PMC8454140; doi:10.1186/s12874-021-01378-8)
Supplement: Supplementary file 1 — Additional file 1. Contains the parameter setting and considerations, as well as the SAS codes, for the simulated mycosis fungoides dataset used in the case study. [file 12874_2021_1378_MOESM1_ESM.pdf]

S1. SAS codes to generate the simulated dataset used in the case study.

1.1 Parameter setting and considerations

The parameters used to generate the simulated data partly came from de Masson's work [1]. Details are explained in the following.

1) The tumor clone frequency (TCF) is a skewed distribution with the median of about 10% for patients with early-stage mycosis fungoides (MF), as shown in Fig.1 in [1]. We generated the variable "TCF0" with a lognormal distribution $\ln(TCF0) \sim N(-2.3, 0.36)$, then a categorical variable "TCF" was generated based on the "TCF0" with TCF=1 if TCF0>25% and TCF=0 otherwise.
2) The median age of the MF patients is about 60 years, as shown in Table S2 in [1]. We generated "age0" following a normal distribution $N(60, 15^2)$ with the maximum being 95 years old. Then a categorical variable "age" was generated based on the "age0" with age=1 if age0>60 and age0=0 otherwise.
3) Assume 70% of patients with early-stage MF are in stage IA and 30% in stage IB. For illustration, the effects of "TCF", "stage", and "age" on the susceptibility to the disease progress were set to be exp(3), exp(2), and exp(1), respectively, according to de Masson's study [1] that "TCF" is a strong predictor of progress, followed by "stage" and "age". The intercept -2.578 was chosen to ensure 80% of patients were insusceptible to the progress.
4) The lifespan for the insusceptible patients was set to be about 80 years (fewer than 100 years) since the indolent MF without disease progress does little to shorten the life expectancy.
5) The effect of "TCF", "stage", and "age" on the progress time and death time were set to be exp(1.6), exp(0.9), and exp(0.7), respectively, according to Table 2 in de Masson's study [1]. The progress and death time for susceptible patients were generated with Weibull distributions and the scale and shape parameters were chosen to make sure that the progress time for susceptible patients was shorter than 10 years and the death time after progress was shorter than 4 years. The effect of the intermediate event "progress" on survival was set to be exp(2) without loss of generality.

1.2 SAS codes

```
data temp;
 format age0 8.1;
  do i=1 to 1000;
    TCF0=exp(-2.3+sqrt(0.36)*normal(123));
    if TCF0>0.25 then TCF=1;else TCF=0;
    stage=rantbl(123,0.7)-1;
    do until (age0<=95);age0=rannor(123)*15+60;end;
    if age0>60 then age=1;else age=0;
    p=1/(1+exp(-(-2.578+3*TCF+2*stage+1*age)));
```

```
        sus=2-rantbl(123,p);
        output;
  end;
run;
data simdata;
    set temp;
    format tdp tdeath 8.1;
        if sus=0 then do;
              tdp=.;
              if age0<80 then do until (tdeath>0 and age1<=100);
                    tdeath=80+rannor(123)*15-age0;
                    age1=age0+tdeath;
              end;
              else do until (age1<=100);
                  tdeath=-log(uniform(123))/0.1;
                  age1=age0+tdeath;
              end;
        end;
        else do;
            tdp=(-log(uniform(123))/(0.1*exp(1.6*TCF+0.9*stage+0.7*age)))**(1/1.5);
            tempv1=-log(uniform(123));
            tempv2=0.05*exp(1.6*TCF+0.9*stage+0.7*age)*(tdp**1.2);
            if tempv1<tempv2 then
                  tdeath=(tempv1/(0.05*exp(1.6*TCF+0.9*stage+0.7*age)))**(1/1.2);
            else tdeath=((tempv1-0.05*exp(1.6*TCF+0.9*stage+0.7*age)*(tdp**1.2))
                          /(0.05*exp(1.6*TCF+0.9*stage+0.7*age+2))+(tdp**1.2))**(1/1.2);
        end;
run;
```

Reference:

1. de Masson A, O'Malley JT, Elco CP, Garcia SS, Divito SJ, Lowry EL, et al. High-throughput sequencing of the T cell receptor beta gene identifies aggressive early-stage mycosis fungoides. Sci Transl Med. 2018;10:eaar5894.
